# Modernizing the U.S. Census Bureau’s Demographic Programs

**DOI:** 10.23889/ijpds.v9i5.2913

**Published:** 2024-09-18

**Authors:** David Johnson

**Affiliations:** 1 Census Bureau

The U.S. Census Bureau is modernizing its approach to producing demographic data to more effectively and efficiently meet the growing demand for high quality, comprehensive, and timely official statistics. This panel introduces four transformation efforts: the Demographic Frame, National Experimental Wellbeing Statistics (NEWS), and the Census Household Panel.

